# Supplementary material for: Lipid-mediated activation of plasma membrane-localized deubiquitylating enzymes modulate endosomal trafficking
Source: Nat Commun. 2022 Nov 12;13:6897. doi: 10.1038/s41467-022-34637-3 (PMC9653390; doi:10.1038/s41467-022-34637-3)
Supplement: Supplementary file 1 — Supplementary Information [file 41467_2022_34637_MOESM1_ESM.pdf]

SUPPLEMENTARY INFORMATION

Lipid-mediated activation of plasma membrane-localized deubiquitylating enzymes modulate endosomal trafficking

Karin Vogel[1], Tobias Bläske[1], Marie-Kristin Nagel[1], Christoph Globisch[2], Shane Maguire[3], Lorenz Mattes[3], Chris Gude[4], Michael Kovermann[5], Karin Hauser[3], Christine Peter[2], and Erika Isono[1*]

[1]Plant Physiology and Biochemistry, Department of Biology, University of Konstanz, Universitätsstraße 10, 78464 Konstanz, Germany

[2]Computational and Theoretical Chemistry, Department of Chemistry, University of Konstanz, Universitätsstraße 10, 78464 Konstanz, Germany

[3]Biophysical Chemistry, Department of Chemistry, University of Konstanz, Universitätsstraße 10, D-78464 Konstanz, Germany

[4]School of Life Sciences, Technical University of Munich, 85354 Freising, Germany

[5]NMR, Department of Chemistry, University of Konstanz, Universitätsstraße 10, 78464 Konstanz, Germany

*e-mail: erika.isono@uni-konstanz.de

Supplemental Table 1: Primers used in this study

primer name	*sequence (5‘-3‘)*
ACT2 fw	ATTCAGATGCCCAGAAGTCTTGTTC
ACT2 rv	GCAAGTGCTGTGATTTCTTTGCTCA
eGFP rv	CTTGTGGCCGTTTACGTCG
CG3 UBP24 GW fw	GGGGACAAGTTTGTACAAAAAAGCAGGCTTGAGTGAAAAGAAGGTATTTG
CG4 UBP24 GW rv	GGGGACCACTTTGTACAAGAAAGCTGGGTTTACACTTGCTTGTAGAAGAGG
CG13 OTU9 GW fw	GGGGACAAGTTTGTACAAAAAAGCAGGCTTGGGGTATGAGCCTGA
CG14 OTU9 GW rv	GGGGACCACTTTGTACAAGAAAGCTGGGTTCAGAATACCCAATATTTCTTC TT
CG15 OTU10 GW fw	GGGGACAAGTTTGTACAAAAAAGCAGGCTTGGTGTCACATGAAGAGAAC
CG16 OTU10 GW rv	GGGGACCACTTTGTACAAGAAAGCTGGGTCTAGTTTCCGAAACGCC
CG17 OTU11 GW fw	GGGGACAAGTTTGTACAAAAAAGCAGGCTTGGATGAAAACCATAGGAAT
CG18 OTU11 GW rv	GGGGACCACTTTGTACAAGAAAGCTGGGTCTAGAAGAGCCAATGCTTC
CG19 OTU12 GW fw	GGGGACAAGTTTGTACAAAAAAGCAGGCTTGGGAGACTCTTCGAGTT
CG20 OTU12 GW rv	GGGGACCACTTTGTACAAGAAAGCTGGGTCTAGAACAACCAATGTTTTCTC
CG25 UBP3 GW fw	GGGGACAAGTTTGTACAAAAAAGCAGGCTTGGGCGCCGC
CG26 UBP3 GW rv	GGGGACCACTTTGTACAAGAAAGCTGGGTTCACTTGGTCGGTCC
CG27 UBP4 GW fw	GGGGACAAGTTTGTACAAAAAAGCAGGCTTGGGCGCGGC
CG28 UBP4 GW rv	GGGGACCACTTTGTACAAGAAAGCTGGGTCTATCTTGTTCCGAGGC
CG46 OTU11 fw BamHI	AAGGGGATCCATGGATGAAAACCATAGGA
CG47 OTU11 rv EcoRI	AAGGGAATTCCTAGAAGAGCCAATGCTTC
CG48 OTU12 fw BamHI	AAGGGGATCCATGGGAGACTCTTCGAGTT
CG49 OTU12 rv XhoI	AAGGCTCGAGCTAGAACAACCAATGTTTTCTCT
FA130 OTU12 GT fw	AGAGTGTCGTTCTATGTATGA
FA131 OTU12 GT rv	ATTCAAGAGATTCTCCACTAAGG
FA134 OTU11 GT fw	CTGATCAGAGGTAGTCTCTG
FA135 OTU11 GT rv	AGGAAGTGAGCTAACTGCTA
KV1 UBP7 GW fw	GGGGACAAGTTTGTACAAAAAAGCAGGCTTGGTGATCAAACCCG
KV2 UBP7 GW rv	GGGGACCACTTTGTACAAGAAAGCTGGGTTTACATGGAGATGAGACG
KV5 UBP9 GW fw	GGGGACAAGTTTGTACAAAAAAGCAGGCTTGACGATCCCTAATTCC
KV6 UBP9 GW rv	GGGGACCACTTTGTACAAGAAAGCTGGGTTCATGATGCTTTGTCATTAT
KV13 UBP22 GW fw	GGGGACAAGTTTGTACAAAAAAGCAGGCTTGTCCGCGAGGAT
KV14 UBP22 GW rv	GGGGACCACTTTGTACAAGAAAGCTGGGTTCAGCAATCAGCAAAG
KV15 UBP23 GW fw	GGGGACAAGTTTGTACAAAAAAGCAGGCTTGGAGGTTGCTACGAG
KV16 UBP23 GW rv	GGGGACCACTTTGTACAAGAAAGCTGGGTTCATATCCTGTAGGCTGTCT
KV17 UBP25 GW fw	GGGGACAAGTTTGTACAAAAAAGCAGGCTTGGGATTTAAACTGCAGAT
KV18 UBP25 GW rv	GGGGACCACTTTGTACAAGAAAGCTGGGTTCACGAGTACTTCTTCTTGC
KV47 OTU11(OTU) fw	AAGGGAATTCTCTGGGAGGTTGG

EcoRI	
KV104 OTU11 N-term SalI rv	AAGGGTCGACACCATATGTGGCCAACC
KV274 SYP121 CDS GW fw	GGGGACAAGTTTGTACAAAAAAGCAGGCTtgAACGATTTGTTTTCCA
KV275 SYP121 CDS GW rv	GGGGACCACTTTGTACAAGAAAGCTGGGTtcaACGCAATAGACGC
KV363 OTU11(OTU) rv SalI	AAGG GTCGAC CCATTAGCGTATAAAGAATTGTAATG
KV380 OTU11 C112A GCC fw	GGAGATGGAAATGCCCAGTTTCGAGCT
KV381 OTU11 C112A GCC rv	AGCTCGAAACTGGGCATTTCCATCTCC
KV417 OTU11(6A2) EcoRI rv	AAGGGAATTCCTAGAAGAGCCAAGCCGCCGCTGCCGGTGCTGCTGTTGGA
KV418 OTU12(6A2) XhoI rv	AAGGCTCGAGCTAGAACAACCAAGCTGCTGCCGCTGGCGCAGCCTGAACT
KV455 OTU12(6A2) GW rv	GGGGACCACTTTGTACAAGAAAGCTGGGTCTAGAACAACCAAGCTGCTGCC GAGCAGC
KV456 OTU11(6A2) rv GW	GGGGACCACTTTGTACAAGAAAGCTGGGTCTAGAAGAGCCAAGCC
KV464 OTU12 C GW fw	GGGGACAAGTTTGTACAAAAAAGCAGGCTTGATGGGAGACTCTTCGAGTT
KV465 OTU12 C GW rv	GGGGACCACTTTGTACAAGAAAGCTGGGTCGAACAACCAATGTTTTCTCTT TG
KV470 OTU11 qRT FW	TGCAAATGCAAGTACAAGTGCTAGAG
KV471 OTU11 qRT RV	CATATGTGGCCAACCTCCCAG
KV472 OTU12 qRT FW	GTTCCACGAATAAATTGTTATATTCCCAACT
KV473 OTU12 qRT RV	AGTTTCCATCACCAGAGACCTTCAA
LH7 OTU12 mut 3xAla2 fw	ACGATATTACGCGGCGATGGGAGCGTTTGGAGAAT
LH8 OTU12 mut 3xAla2 rv	ATTCTCCAAACGCTCCCATCGCCGCGTAATATCGT
LH9 OTU11(6A1) mutagenesis fw	TATGTGCCAATGGCGTACGCGCACTACACCGCGGCGATGGCG
LH10 OTU11(6A1) mutagenesis rv	CGCCATCGCCGCGGTGTAGTGCGCGTACGCCATTGGCACATA
LH11 OTU12(6A1) mutagenesis fw	TGTTCCGATGGCGTACGCGGCGTATTACGCGG
LH12 OTU12(6A1) mutagenesis rv	CCGCGTAATACGCCGCGTACGCCATCGGAACA
TB1 GG OTU11P fw	ATATGGTCTCAGCGGCGTTAAACCGAGTGTGGCAAGAGG
TB5 GG OTU11P rv	ATATGGTCTCTCAGATTTGGATCCTATACTAAATACAACCTAGGAAGC
TB6 GG OTU11 fw	ATATGGTCTCATCTGTACAATGGATGAAAACCATAGGAATCC
TB12 GG OTU11 fw	ATATGGTCTCACACCATGGATGAAAACCATAGGAATCCATTTG
TB13 GG OTU11 rv	ATATGGTCTCTCCTTCTAGAAGAGCCAATGCTTCCTTCTCG
TB14 GG OTU11T fw	ATATGGTCTCAAAGGTATTGGATTTAAAGTTCATCTCCATTTGTA
TB15 GG OTU11T rv	ATATGGTCTCTGATTCGATCCCATCCCACTGATTC
TB18 GG OTU11 rv	ATATGGTCTCTGGTGCCGAAGAGCCAATGCTTCCTTCTCG
TB23 GG OTU12P rv	ATATGGTCTCTCAGAGTTTAAAAGCTCGTTCCTTACTAATTG
TB24 GG OTU12 fw	ATATGGTCTCATCTGTACAATGGGAGACTCTTCGAGTTCAAC
TB25 GG OTU12 rv	ATATGGTCTCTGGTGCCGAACAACCAATGTTTTCTCTTTGGC

TB26 GG OTU12 fw	ATATGGTCTCACACCATGGGAGACTCTTCGAGTTCAAC
TB27 GG OTU12 rv	ATATGGTCTCTCCTTCTAGAACAACCAATGTTTTCTCTTTGG
TB28 GG OTU12T fw	ATATGGTCTCAAAGGAAGGCACGCCACCATACATGTAT
TB29 GG OTU12T rv	ATATGGTCTCTGATTCAATGATGCTCTCTGTTGCTCTC
TB32 mut OTU11 fw	GAAAACATCTCTGAGATCGACGAAAC
TB33 mut OTU11 rv	GTTTCGTCGATCTCAGAGATGTTTTC
TB37 mut OTU12a fw	GTGAGTTGAAGGACTCTGGTG
TB38 mut OTU12a rv	CACCAGAGTCCTTCAACTCAC
TB39 mut OTU12b fw	GAGAATGGGGAGTCCATATTAC
TB40 mut OTU12b rv	GTAATATGGACTCCCCATTCTC
TB320 GG OTU12P fw	ATATGGTCTCAGCGGCAGTCCCTCCACCTGTCTTGAC
TB448_OTU11-6A1-f	CGGCGATGGCGAAACATGGTGAATG
TB449_OTU11-6A1-r	CATTCACCATGTTTCGCCATCGCCG
MN439 A fw U6-26 P	ATATGGTCTCAGCGGCGACTTGCCTTCCGCA
MN440 B rv U6-26 T	ATATGGTCTCTCAGATATTGGTTTATCTCATCGGAACTG
MN445 D fw CAS9-NLS-FLAG	ATATGGTCTCAAAGGAACAATGGACTATAAGGACCACGAC
MN446 E rv CAS9	ATATGGTCTCTGATTTCACTTCTTCTTCTTCGCCT
MN457 OTU12 target U6-26 rv	CACCGTCTAGTTTAGAGTACAATCACTACTTCGACTCTAGC
MN458 OTU12 target gRNA fw	GTACTCTAAACTAGACGGTGGTTTTAGAGCTAGAAATAGCAAGTT
MN516 OTU12 mut U6-26 rv	TATCGTCTGACTTAGAGTACAATCACTACTTCGACTCTAGC
MN517 OTU12 mut gRNA fw	GTACTCTAAGTCAGACGATAGTTTTAGAGCTAGAAATAGCAAGTT

Supplemental Table 2: Plasmids used in this study

plasmid name	*description*	*vector backbone*	*source*
pCG19	*35Spro:YFP-UBP9*	pExTag-YFP (MPI Cologne)	this study
pCG20	*35Spro:YFP-UBP24*	pExTag-YFP (MPI Cologne)	this study
pCG22	*35Spro:YFP-OTU12*	pExTag-YFP (MPI Cologne)	this study
pKV9	*35Spro:YFP-UBP4*	pExTag-YFP (MPI Cologne)	this study
pKV10	*35Spro:YFP-UBP6*	pExTag-YFP (MPI Cologne)	this study
pKV11	*35Spro:YFP-UBP10*	pExTag-YFP (MPI Cologne)	this study
pKV12	*35Spro:YFP-UBP12*	pExTag-YFP (MPI Cologne)	this study
pKV13	*35Spro:YFP-UBP18*	pExTag-YFP (MPI Cologne)	this study
pKV14	*35Spro:YFP-UBP20*	pExTag-YFP (MPI Cologne)	this study
pKV15	*35Spro:YFP-UBP27*	pExTag-YFP (MPI Cologne)	this study
pKV17	*35Spro:YFP-UBP3*	pExTag-YFP (MPI Cologne)	this study
pKV19	*35Spro:YFP-UBP7*	pExTag-YFP (MPI Cologne)	this study
pKV20	*35Spro:YFP-UBP25*	pExTag-YFP (MPI Cologne)	this study
pKV22	*35Spro:YFP-UBP23*	pExTag-YFP (MPI Cologne)	this study
pKV24	*35Spro:YFP-UBP22*	pExTag-YFP (MPI Cologne)	this study

pKV26	*GST-OTU12 (FL)*	pGEX-6P1 (Sigma Aldrich)	this study
pKV30	*35Spro:GFP-OTU11*	pFASTR06 [6]	this study
pKV31	*35Spro:GFP-OTU12*	pFASTR06 [6]	this study
pKV64B	*35Spro:YFP-OTU9*	pExTag-YFP (MPI Cologne)	this study
pKV65B	*35Spro:YFP-OTU10*	pExTag-YFP (MPI Cologne)	this study
pKV67	*GST-OTU11 (N)*	pGEX-6P1 (Sigma Aldrich)	this study
pKV118	*GST-OTU11.1*	pGEX-6P1 (Sigma Aldrich)	this study
pKV119	*GST-OTU11.2*	pGEX-6P1 (Sigma Aldrich)	this study
pKV137	*35Spro:RFP-SYP121*	pExTag-RFP	this study
pKV168	*35Spro:GFP-OTU11 (6A1)*	pFASTR06 [6]	this study
pKV172	*35Spro:GFP-OTU12 (6A1)*	pFASTR06 [6]	this study
pKV185	*GST-OTU11(OTU-6A1)*	pGEX-6P1 (Sigma Aldrich)	this study
pKV186	*GST-OTU11(OTU)*	pGEX-6P1 (Sigma Aldrich)	this study
pKV190	*35Spro:RFP-OTU11 (6A1)*	pExTag-RFP	this study
pKV195	*GST-OTU11 (C112A)*	pGEX-6P1 (Sigma Aldrich)	this study
pKV214	*35Spro:RFP-OTU12*	pExTag-RFP	this study
pKV216	*35Spro:RFP-OTU12(6A1)*	pExTag-RFP	this study
pKV230	*GST-OTU12(6A1)*	pGEX-6P1 (Sigma Aldrich)	this study
pKV236	*GST-OTU12(6A2)*	pGEX-6P1 (Sigma Aldrich)	this study
pKV243	*GST-OTU11(6A2)*	pGEX-6P1 (Sigma Aldrich)	this study
pKV246	*GST-OTU11(6A1+6A2)*	pExTag-YFP (MPI Cologne)	this study
pKV255	*35Spro:YFP-OTU11*	pExTag-YFP (MPI Cologne)	this study
pKV258	*35Spro:YFP-OTU12(6A2)*	pExTag-YFP (MPI Cologne)	this study
pKV261	*35Spro:YFP-OTU12(6A2)*	pExTag-YFP (MPI Cologne)	this study
pKV263	*35Spro:OTU12-RFP*	35S-GW-RFP (MPI Cologne)	this study
pKV265	*GST-OTU11(6A1)*	pGEX-6P1 (Sigma Aldrich)	this study
pKV266	*35Spro:YFP-OTU11(6A1)*	pExTag-YFP (MPI Cologne)	this study
pKV268	*35Spro:YFP-OTU12(6A1)*	pExTag-YFP (MPI Cologne)	this study
pMN179	*CRISPR^{OTU12}*	pUC57 (Thermo Fisher Scientific)	this study
pMN187	*CRISPR^{OTU12m}*	pUC57 (Thermo Fisher Scientific)	this study
pTB39	*OTU11pro:GFP-genomic OTU11*	pBB10	this study
pTB114	*OTU12pro:GFP-genomic OTU12*	pBB10	this study

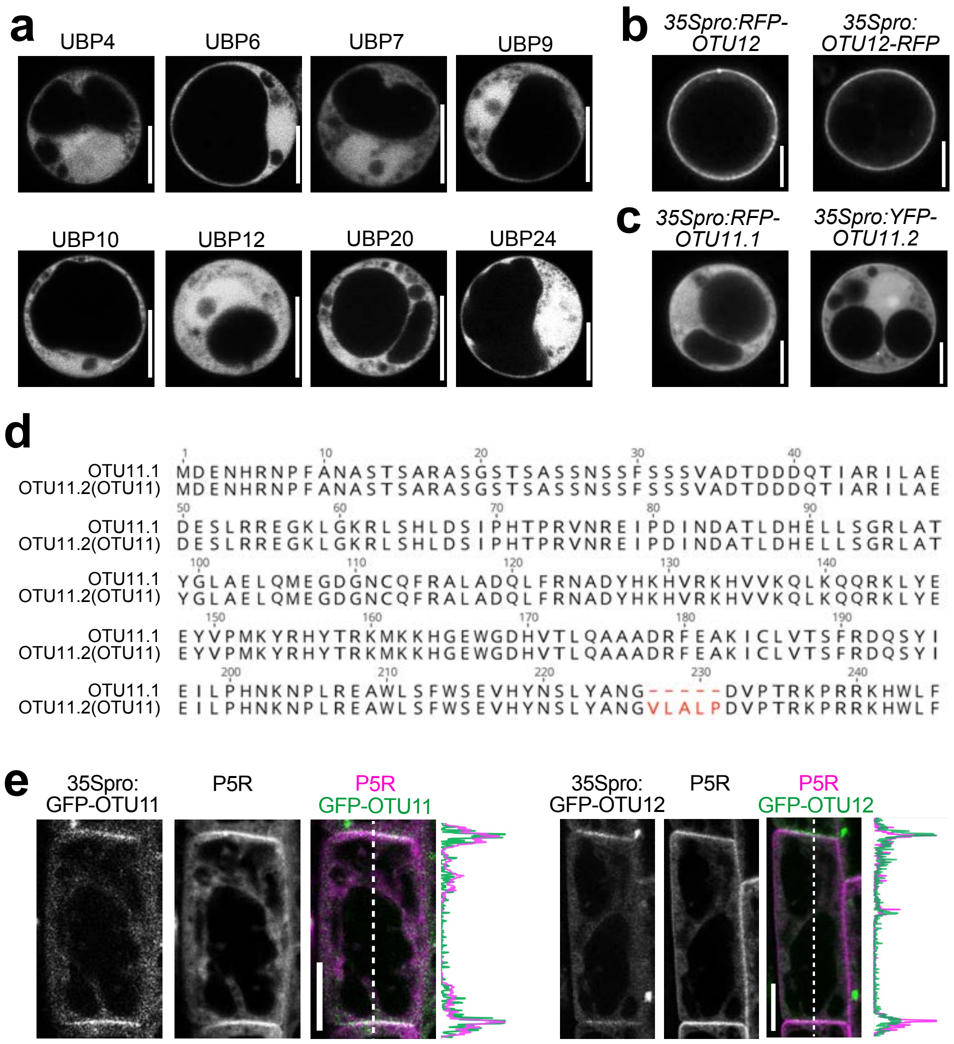

Supplementary Figure 1: Localization of YFP-fused Arabidopsis DUBs

(a) Representative confocal pictures of YFP-fused UBP4, UBP6, UBP7, UBP9, UBP10, UBP12, UBP20, and UBP24. 35S promotor-driven YFP-fusion constructs were transiently overexpressed in Arabidopsis root cell-derived protoplasts and analyzed under a confocal microscope. Scale bars: 10 µm.

(b) Both N- and C-terminal fusion of OTU12 localize to the PM. Representative confocal microscopy images of Arabidopsis cell culture-derived protoplasts transformed with 35Spro:RFP-OTU12 and 35Spro:OTU12-RFP. Scale bars: 10 µm.

(c) Splicing variations of OTU11 do not affect the localization. Representative confocal microscopy images of Arabidopsis cell culture-derived protoplasts expressing *35Spro:RFP-OTU11.1* and *35Spro:YFP-OTU11.2*. Scale bars: 10 µm.

(d) Alignment of OTU11.1 and OTU11.2 (referred to as OTU11 in the manuscript). The splicing variant OTU11.2 has five additional amino acids (aa 227 to 231).

(e) Confocal images of 35Spro:GFP-OTU11 and 35Spro:GFP-OTU12 lines co-expressing the PI(4)P-biosensor P5R. Both GFP-OTU11 (upper panel) and GFP-OTU12 (lower panel) co-localize with P5R at the PM. The intensity profiles along the middle of the overlay images (dotted lines) are shown at the right side of the panels. Scale bars: 10 µm.

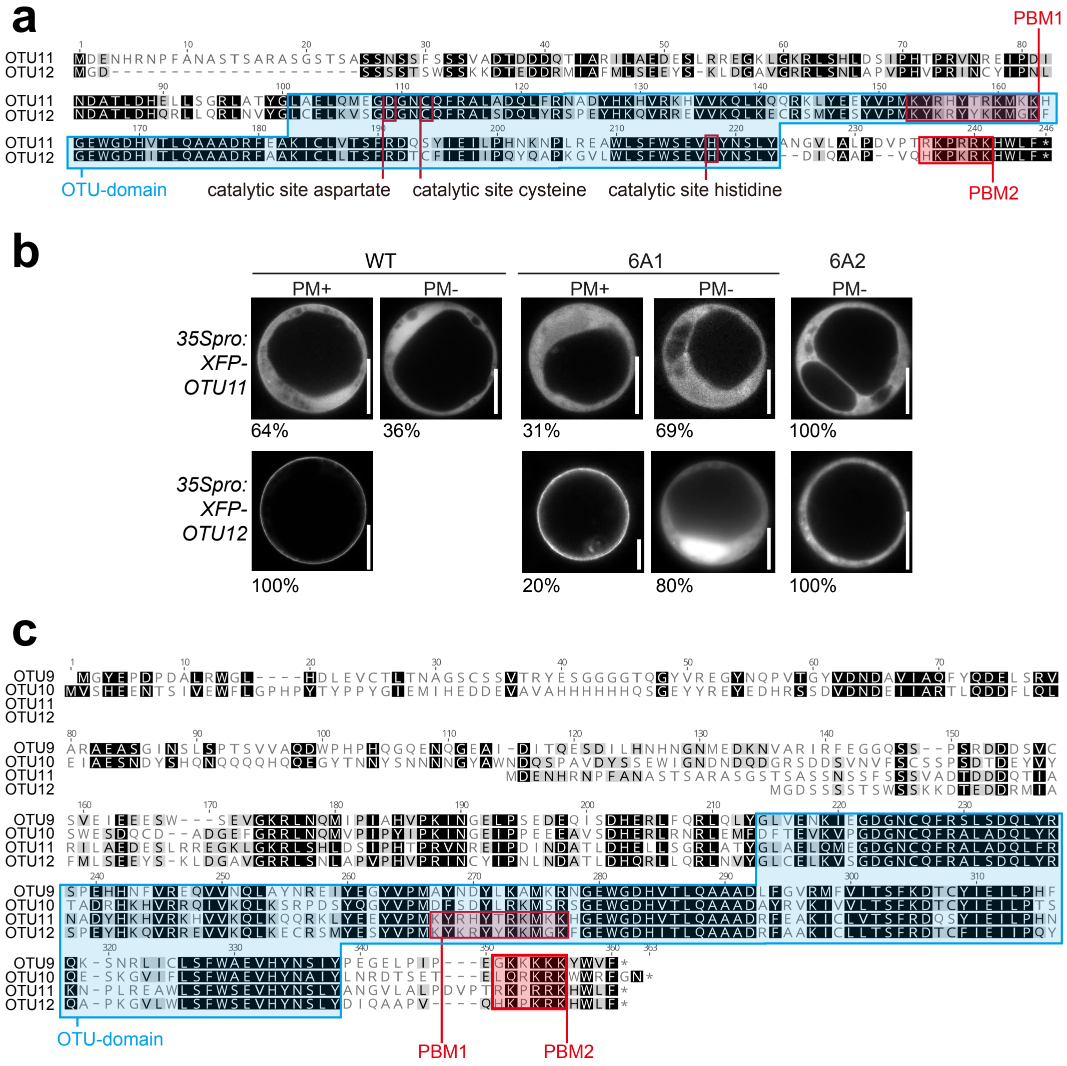

Supplementary Figure 2: Amino acid alignment of OTU DUBs

(a) Alignment of the amino acid sequences of OTU11 and OTU12. The OTU domain is highlighted in blue, the active site cysteine, aspartate, histidine, PBM1 and PBM2 in red. Black; conserved amino acids, dark gray; amino acids with similar properties.

(b) Representative images of protoplasts after transformation with the indicated XFP-fusion constructs of OTU11 and OTU12 analyzed in Figure 2e and f. *35Spro:RFP-OTU11(WT)* (n=22), *35Spro:Y/RFP-OTU11(6A1)* (n=32), *35Spro:YFP-OTU11(6A2)* (n=20), *35Spro:YFP-OTU12(WT)* (n=77), *35Spro:YFP-OTU12(6A1)* (n=56), and *35Spro:YFP-OTU12(6A2)* (n=15) expressing protoplasts were analyzed. The percentage of each localization pattern is indicated for each experiment. The transformation was conducted at least twice for each construct. Scale bars: 10 µm

(c) Alignment of the amino acid sequences of OTU9, OTU10, OTU11 and OTU12. Note that whereas PBM1 is specific to OTU11 and OTU12, PBM2 is conserved. Black; conserved amino acids, dark gray; amino acids with similar properties, light gray; amino acids conserved in part of the sequences.

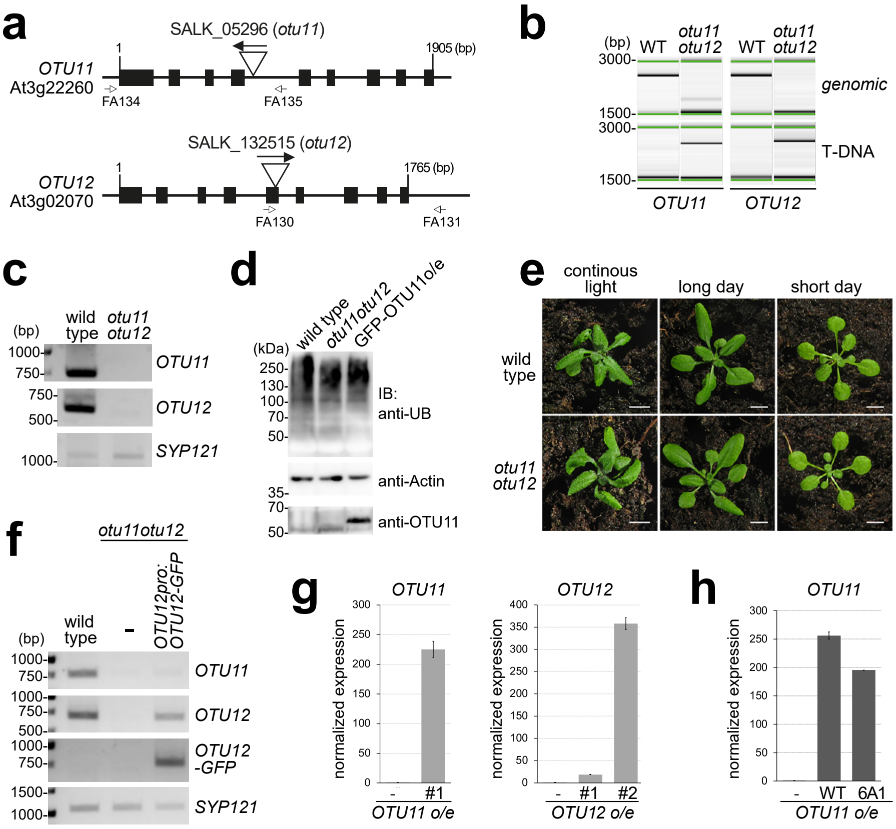

Supplementary Figure 3: *otu11otu12* does not show apparent defects in growth and development.

(a) Scheme showing the intron and exon structure of *OTU11*(At3g22260) and *OTU12* (At3g02070) and the position of the T-DNA insertions. In the *otu11* line (SALK_05296), the insertion is positioned in the fifth intron shortly after the fourth exon, in the *otu12* line (SALK_132515) the insertion is positioned in the fifth exon. The position of the genotyping primers FA134/FA135 (*OTU11*) and FA130/FA131 (*OTU12*) is indicated with arrows with un-filled arrowheads.

(b) Genotyping-PCR of wild-type and *otu11otu12* seedlings. PCR products for genomic fragments and T-DNA-containing fragments were analyzed by capillary electrophoresis. Source data are provided as a Source Data file.

(c) RT-PCR of wild-type and *otu11otu12* seedlings. cDNA of wild-type and *otu11otu12* seedlings were analyzed by PCR using gene-specific primers for *OTU11*, *OTU12*, and *SYP121* (loading control). Source data are provided as a Source Data file.

(d) Immunoblotting with an anti-ubiquitin antibody on total plant extracts of 10-day old wild-type, *otu11otu12* and *35Spro:GFP-OTU11* seedlings. An anti-ACTIN antibody was used as a loading control on the same membrane as the anti-OTU11 blot. Source data are provided as a Source Data file.

(e) Photographs of 4-week-old wild-type and *otu11otu12* plants grown under continuous light, long day (16 h light/8 h dark), and short day (8 h light/16 h dark) conditions on soil. Scale bars: 1 cm.

(f) RT-PCR of wild type, *otu11otu12*, and *otu11otu12* containing *OTU12pro:OTU12-GFP*. Total RNA was extracted from 7-day-old seedlings, and cDNA was analyzed by PCR using gene-specific primers for *OTU11*, *OTU12*, *OTU12-GFP*, and *SYP121* (loading control). Source data are provided as a Source Data file.

(g)(h) qRT-PCR of *OTU11(WT)*- (g, h) , *OTU12(WT)*- (g) and *OTU11(6A1)*-overexpressing lines (h). Total RNA was extracted from 7-day-old seedlings, and cDNA was analyzed by qRT-PCR using gene-specific primers. Normalized expression-levels of OTU11 and OTU12 are shown for one *35Spro:GFP-OTU11(WT)* line, two *35Spro:GFP-OTU12 (WT)* lines and one *35Spro:GFP-OTU11(6A1)* line. The expression was normalized against *ACTIN2* and the expression level of wild type was set to 1. The analysis was carried out three times each with technical quadruplicates (g) or triplicates (h) and a representative result is shown. Error bars indicate the standard error of mean of the technical replicates. Source data are provided as a Source Data file.

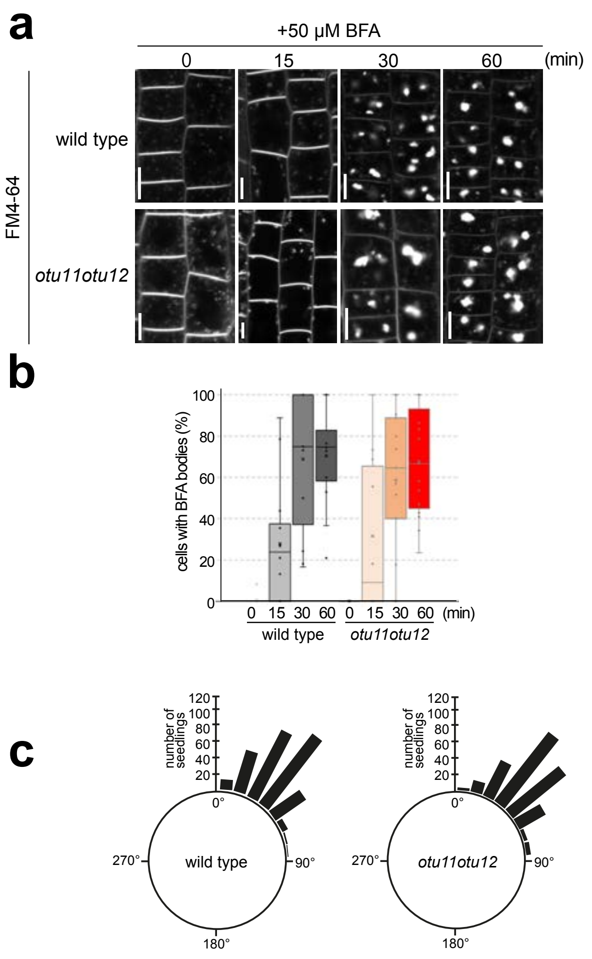

Supplementary Figure 4: *otu11otu12* cells do not show apparent changes in the accumulation of FM4-64 to BFA bodies.

(a) Representative images of wild-type and *otu11otu12* seedlings treated with 2 µM FM4-64 and 50 µM brefeldin A fort he indicated time. Scale bars: 10 µm

(b) Boxplot of the quantification results in (a). Center line, median; box limits, first and third quartiles; whiskers, 1.5x interquartile range; points, outliers. The number of cells with FM4-64 signals with BFA bodies. BFA bodies with sizes >2000 (a.u.) were counted for each time point and then the percentage of cells with BFA bodies was calculated for each seedling. The experiment was conducted three times. A two-tailed *t*-test with no equal variance was performed. The differences between wild type and otu11otu12 were not significant (n.s. p>0.5) at all analyzed time points. wild type/ *otu11otu12* 15 min (p=0.811, wild type number of seedlings (n)=12, 358 cells, *otu11otu12* n=10, 225 cells), 30 min (p=0.495, wild type n=11, 385 cells, *otu11otu12* n=13, 282 cells), 60 min (p=0.778, wild type n=12, 282 cells, *otu11otu12* n=15, 309 cells). Source data are provided as a Source Data file.

(c) Gravitropism assay with wild-type and *otu11otu12* seedlings. 5-day-old vertically grown seedlings were rotated 90˚, the angle between the gravity direction and the tip of the primary root was measured after 2 hrs and subtracted from 180°. The experiment was repeated twice and the result of one experiment is shown (wild type n=298 seedlings, *otu11otu12* n=239 seedlings). p=0.0986 (p>0.05, n.s., two-tailed *t*-test, no equal variance). Source data are provided as a Source Data file.

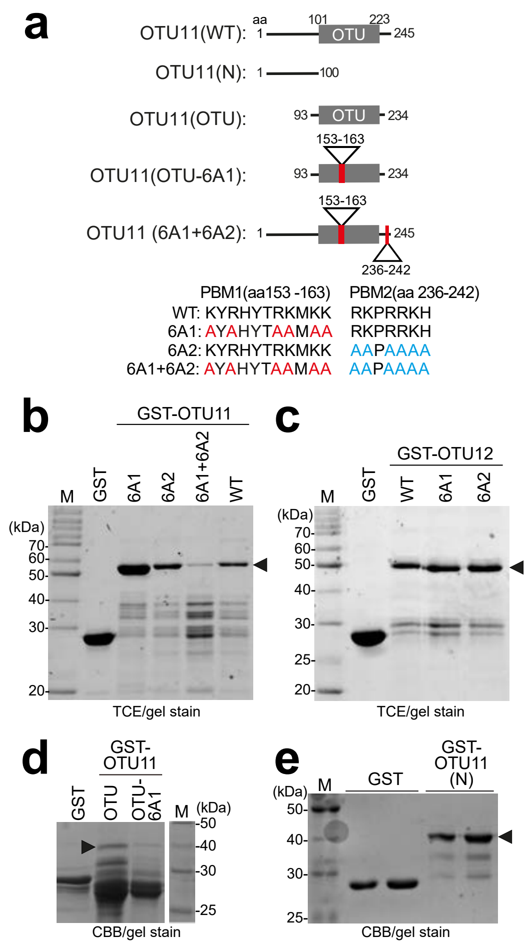

Supplementary Figure 5: Recombinant proteins used for the lipid overlay assay.
(a) Schemes of constructs used for the lipid-binding studies. In addition to constructs described in Figure 2a, the shown construcs were used.
(b)(c)(d)(e) Proteins gels of purified OTU11 variants (b), OTU12 variants (c), and OTU11 fragments (d and e) used for the lipid overlay assays in Figure 5. Protein bands were stained with TCE or CBB as indicated, quantified using the Benchmark^{TM} protein ladder (M, marker) as a standard and used for the lipid overlay assay. Arrowheads indicate the position of OTU11- (b) and OTU12 variants (c). The arrowhead in (d) indicates the position of OTU11(OTU) variants and the arrowhead in (e) indicates the position of OTU11(N). Source data are provided as a Source Data file.

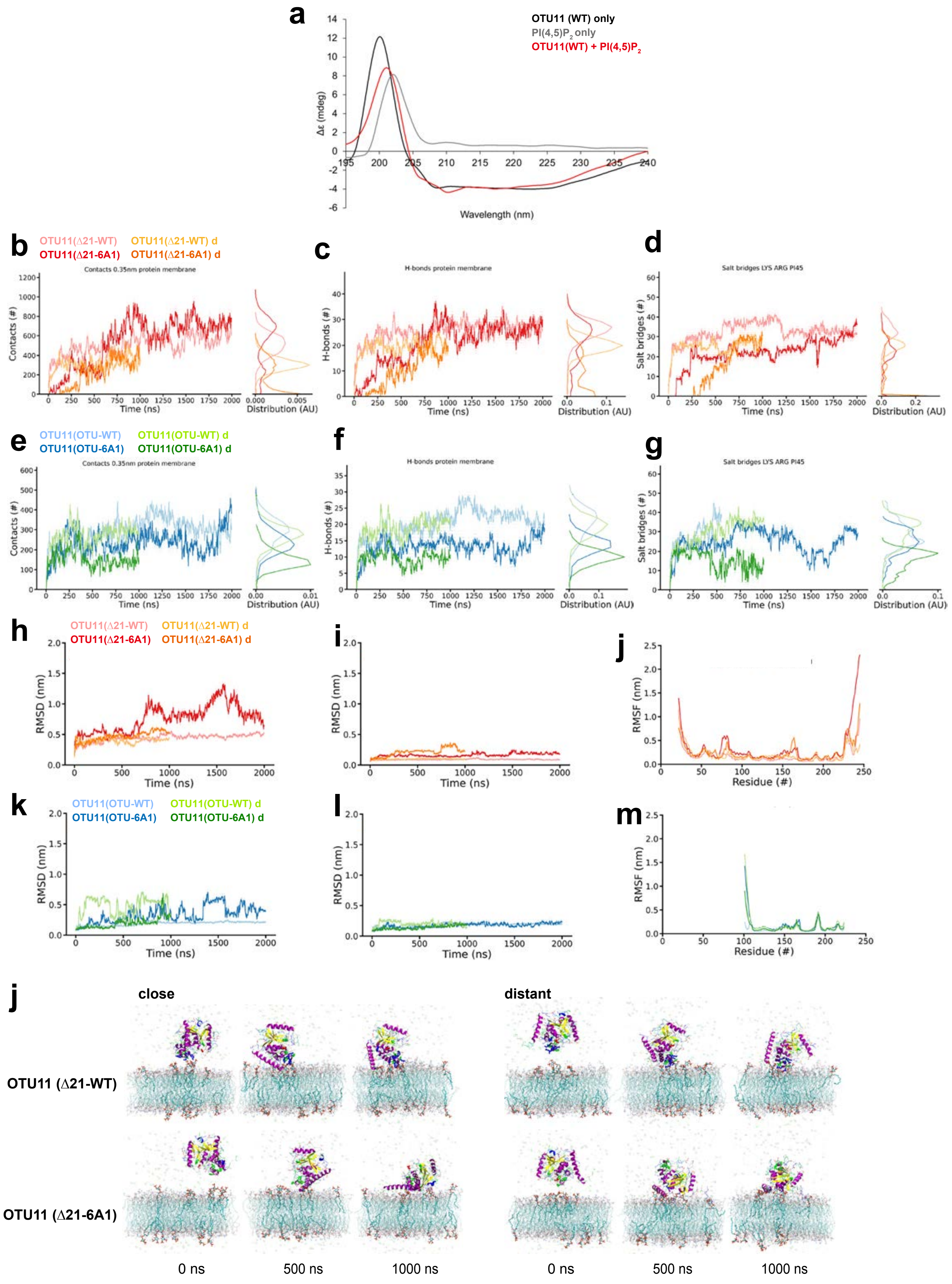
a
OTU11 (WT) only
PI(4,5)P2 only
OTU11(WT) + PI(4,5)P2
Δε (mdeg)
Wavelength (nm)
b
OTU11(Δ21-WT)
OTU11(Δ21-6A1)
OTU11(Δ21-WT) d
OTU11(Δ21-6A1) d
Contacts 0.35nm protein membrane
Contacts (#)
Time (ns)
Distribution (AU)
c
H-bonds protein membrane
H-bonds (#)
d
Salt bridges LYS ARG PI45
Salt bridges (#)
e
OTU11(OTU-WT)
OTU11(OTU-6A1)
OTU11(OTU-WT) d
OTU11(OTU-6A1) d
f
g
h
RMSD (nm)
i
j
RMSF (nm)
Residue (#)
k
l
m
j
close
distant
OTU11 (Δ21-WT)
OTU11 (Δ21-6A1)
0 ns
500 ns
1000 ns

Supplementary Figure 6: Phospholipid binding could induce conformational changes in OTU11.

(a) CD spectra of OTU11 (black), liposomes containing PI(4,5)P_2 (gray) and OTU11 with PI(4,5)P_2-containing liposomes (red). Six scans were accumulated at a scanning speed of 100 nm/s and a wavelength interval of 0.1 nm. Spectra were recorded from 180 nm to 300 nm and are the average of 3 to 6 independent measurements. The protein spectrum shows a-helical structure with the characteristic minima at 208 nm and 222 nm. Secondary structure analysis was performed with DichroWeb.

(b)(c) Interactions between the OTU11(Δ21) (b) and OTU11(OTU) (c) and the lipid bilayer. Number of contacts (atoms pairs below 0.35 nm distance) between the protein and the lipids including all kinds of interactions between both partners (left panels), number of hydrogen bonds between the protein and the lipids (middle panels), number of salt bridges between the positively charged lysine and arginine sidechains with the phosphate groups of phosphoinositol lipids (right panels). “d” indicates simulation with larger initial distance.

(d)(e) Structural deviation and fluctuation of C-alpha atom positions during the simulations of OTU11(Δ21) (d) and OTU11(OTU) (e). Root mean square deviation (RMSD) values of C-alpha atoms with respect to the first frame of the production run (left panels). RMSD values of the OTU-Domain part without the first 7 amino acids (residue 108 to 223) indicating the general stability of folded part of the OTU domain in the models (middle panels). Root mean square fluctuation (RMSF) values for the C-alpha atoms (right panels).

(f) Snapshots from the simulations. Three representative snapshots from the simulations of OTU11(Δ21-WT) and OTU11(Δ21-6A1) showing the starting system, a frame from the middle off the simulation and the final step. The protein is represented as new ribbon and colored according to the secondary structure. Additionally, side chains represented as lines and colored according to the residue type (blue basic, red acidic, green polar, white hydrophobic). The atoms of the catalytic center and the PBM1 motif are highlighted as ball and sticks. The POPC and POPE lipids are depicted as lines and colored according to the atom type. PI(4,5)P_2 are highlighted in licorice representation. The ions are shown as transparent spheres. OTU11(Δ 21-6A1) shows an alternative binding to the membrane in which the binding to the lipids through PBM1 is replaced by PBM2 and the N-terminal helix.

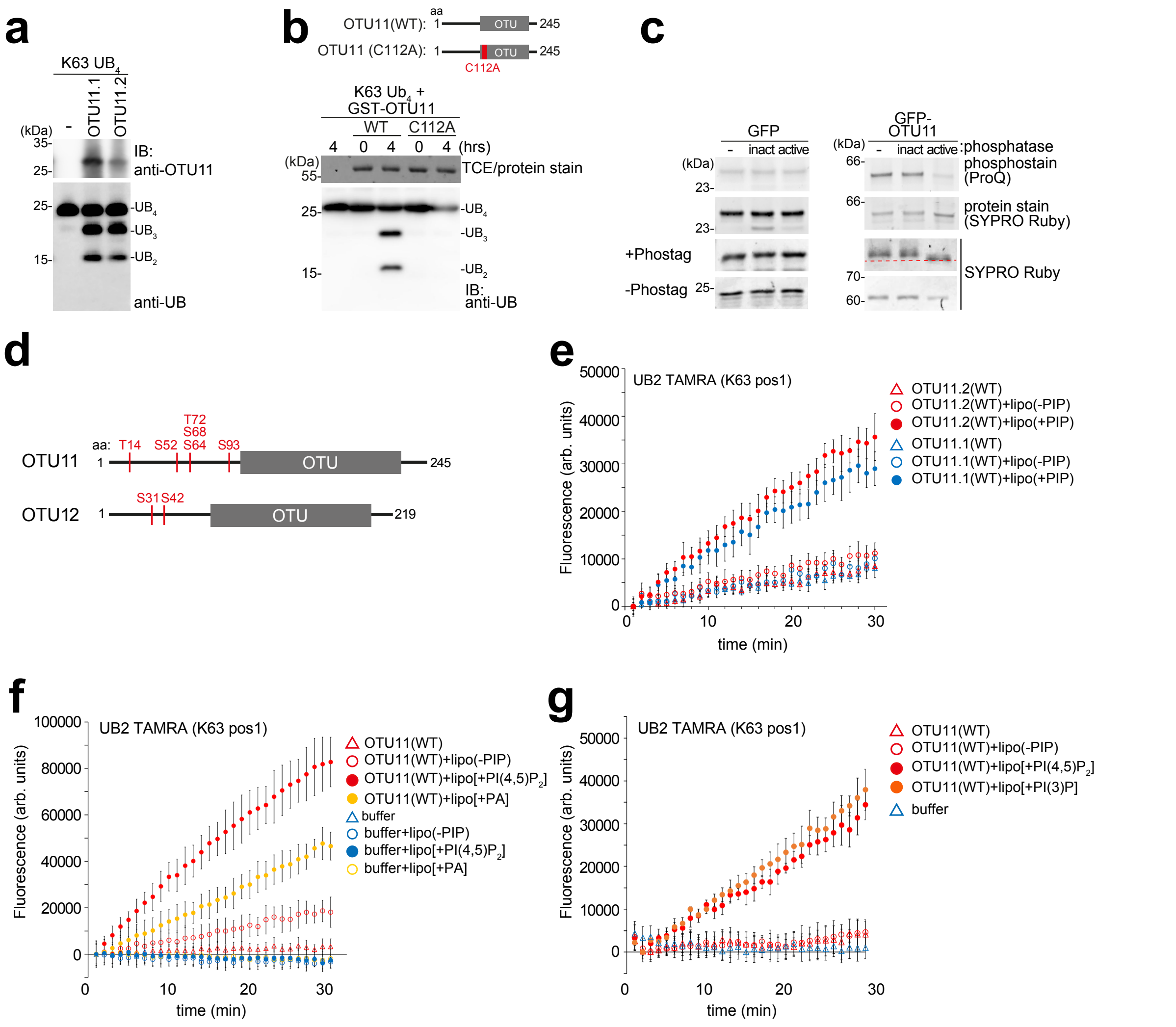

Supplementary Figure 7: OTU11 is activated by the binding to different anionic lipids *in vitro.*
(a) *In vitro* DUB assay with 60 pmol of OTU11.1 and OTU11.2, and 8 pmol of K63-linked tetra-UB, incubated for 2 hours at 21°C. No obvious difference in the cleavage efficiency between the two isoforms could be observed.
(b) *In vitro* DUB assay with 40 pmol of GST-OTU11(WT) or GST-OTU11(C112A), incubated with 7.5 pmol of K63-linked tetra-UB (substrate:enzyme 1:5) for 4 hours at 21°C. Source data are provided as a Source Data file.
(c) OTU11 is phosphorylated in planta. GFP and GFP-OTU11 were purified from 10-day-old Arabidopsis seedlings and incubated with heat-inactivated or active λ-phosphatase for 1 hour at 30°C. The phosphorylation status was analyzed with a phosphostain (ProQ Diamond) or a PhosTag-acrylamide gel (+Phostag). The same gel used for phosphostain was stained with SYPRO Ruby to stain all proteins. The red dotted line indicates the position of unphosphorylated GFP-OTU11. Active: active phosphatase, inact: heat-inactivated phosphatase. Source data are provided as a Source Data file.
(d) A scheme showing the potential phosphorylation sites in the amino acid sequence of OTU11 (T14, S52, S64, S68, T72 and S93) and OTU12 (S31, S42).
(e)(f)(g) DUB assay with a fluorogenic substrate using two splicing variations of OTU11 (e), or with OTU11.2 and liposomes containing PA (f) or PI(3)P (g). 3.75 pmol of OTU11.1 or OTU11.2 was preincubated for 15 minutes with liposomes with [lipo(+PIP)] or without $PI(4,5)P_2$ [lipo(-PIP)] (e), or with liposomes containing the indicated lipids (f, g) before the addition of 3.75 pmol of di-UB (K63-linked) FRET TAMRA Pos1. The fluorescence was measured every minute for 30 minutes. The experiments were conducted at least three times each with technical quadruplicates. A representative result is shown with error bars indicating the standard deviation of technical replicates with the mean as the center of the error bars. Source data are provided as a Source Data file.
