# Supplementary material for: Lipid-mediated activation of plasma membrane-localized deubiquitylating enzymes modulate endosomal trafficking
Source: Nat Commun. 2022 Nov 12;13:6897. doi: 10.1038/s41467-022-34637-3 (PMC9653390; doi:10.1038/s41467-022-34637-3)
Supplement: Supplementary file 3 — Description of Additional Supplementary Files [file 41467_2022_34637_MOESM3_ESM.pdf]

Description of Additional Supplementary Files

File Name: Supplementary Movie 1

Description: Simulation with OTU11(Δ21-WT) placed distantly to the lipid bilayer containing PI(4,5)P2. The protein is represented as new ribbon and colored according to the secondary structure. Side chains represented as lines and colored according to the residue type (blue basic, red acidic, green polar, white hydrophobic). The atoms of the catalytic center and the PBM1 motif are highlighted as ball and sticks. The POPC and POPE lipids are depicted as lines and colored according to the atom type. PI(4,5)P2 molecules are highlighted in licorice representation. The ions are shown as transparent spheres.

File Name: Supplementary Movie 2

Description: Simulation with OTU11(Δ21-6A1) placed distantly to the lipid bilayer containing PI(4,5)P2. The protein is represented as new ribbon and colored according to the secondary structure. Side chains represented as lines and colored according to the residue type (blue basic, red acidic, green polar, white hydrophobic). The atoms of the catalytic center and the PBM1 motif are highlighted as ball and sticks. The POPC and POPE lipids are depicted as lines and colored according to the atom type. PI(4,5)P2 molecules are highlighted in licorice representation. The ions are shown as transparent spheres. OTU11(Δ21-6A1) of shows an alternative binding to the membrane without the involvement of PBM1.

File Name: Supplementary Data 1

Description: PDB-Files of the initial and final configurations of the molecular dynamics simulations of OTU11 Δ21 WT.

File Name: Supplementary Data 2

Description: Description:

PDB-Files of the initial and final configurations of the molecular dynamics simulation of OTU11 Δ21 6A1.

File Name: Supplementary Data 3

Description: PDB-Files of the initial and final configurations of the molecular dynamics simulation of OTU11 OTU WT.

File Name: Supplementary Data 4

Description: PDB-Files of the initial and final configurations of the molecular dynamics simulation of OTU11 OTU 6A1.
