# Supplementary material for: Lipid-mediated activation of plasma membrane-localized deubiquitylating enzymes modulate endosomal trafficking
Source: Nat Commun. 2022 Nov 12;13:6897. doi: 10.1038/s41467-022-34637-3 (PMC9653390; doi:10.1038/s41467-022-34637-3)
Supplement: Supplementary file 10 — Reporting Summary [file 41467_2022_34637_MOESM10_ESM.pdf]

nature portfolio

Corresponding author(s): Erika Isono

Last updated by author(s): Oct 24, 2022

Reporting Summary

Nature Portfolio wishes to improve the reproducibility of the work that we publish. This form provides structure for consistency and transparency in reporting. For further information on Nature Portfolio policies, see our Editorial Policies and the Editorial Policy Checklist.

Statistics

For all statistical analyses, confirm that the following items are present in the figure legend, table legend, main text, or Methods section.

n/a	Confirmed	
☐	☒	The exact sample size (n) for each experimental group/condition, given as a discrete number and unit of measurement
☐	☒	A statement on whether measurements were taken from distinct samples or whether the same sample was measured repeatedly
☐	☒	The statistical test(s) used AND whether they are one- or two-sided<br>*Only common tests should be described solely by name; describe more complex techniques in the Methods section.*
☒	☐	A description of all covariates tested
☒	☐	A description of any assumptions or corrections, such as tests of normality and adjustment for multiple comparisons
☐	☒	A full description of the statistical parameters including central tendency (e.g. means) or other basic estimates (e.g. regression coefficient) AND variation (e.g. standard deviation) or associated estimates of uncertainty (e.g. confidence intervals)
☐	☒	For null hypothesis testing, the test statistic (e.g. F, t, r) with confidence intervals, effect sizes, degrees of freedom and P value noted<br>*Give P values as exact values whenever suitable.*
☒	☐	For Bayesian analysis, information on the choice of priors and Markov chain Monte Carlo settings
☒	☐	For hierarchical and complex designs, identification of the appropriate level for tests and full reporting of outcomes
☒	☐	Estimates of effect sizes (e.g. Cohen's d, Pearson's r), indicating how they were calculated

Our web collection on statistics for biologists contains articles on many of the points above.

Software and code

Policy information about availability of computer code

Data collection	GROMACS version 2021.4 [https://www.gromacs.org], LINCS algorithm (GROMACS), CHARMM36m [http://mackerell.umaryland.edu/charmm_ff.shtml], CHARMM-GUI membrane builder 3.7 [https://charmm-gui.org/?doc=tutorial&project=membrane], The Leapfrog Integrator [http://www.physics.drexel.edu/~steve/Courses/ Comp_Phys/Integrators/leapfrog/], Amersham Imager 600 1.2.0 (Cytiva), Typhoon scanner FLA9500 (Cytiva), ZEN Black 2.3 (Zeiss), Bio-Rad CFX Maestro 1.1 (Bio-Rad), Tecan i-control 2.0 (Tecan), Tecan Magellan Pro 7.3 (Tecan)
Data analysis	Excel 2016 (Microsoft), Fiji 2.9.0 [https://imagej.net/software/fiji/], Amersham Imager analysis software 1.0 (Cytiva), BH-search [https://hpcwebapps.cit.n ih. gov/ bhsearch/], DichroWeb [http://dichroweb.cryst.bbk.ac.uk /html/home.shtml], GROMACS version 2021.4 [https://www. gromacs.org], MDAnalysis 2.0.0 [https://www.mdanalysis.org], Alphafold 2022-06-01 [https://alphafold.ebi.ac.uk], UCSF Chimera 1.16 [https://www.cgl.ucsf.edu/chimera/], VMD 1.9.3 [http://www.ks.uiuc.edu/Research/vmd/], Pymol 2.0.2]https://pymol.org/2/], Photoshop 2020 (Adobe), Illustrator 2020 (Adobe)

For manuscripts utilizing custom algorithms or software that are central to the research but not yet described in published literature, software must be made available to editors and reviewers. We strongly encourage code deposition in a community repository (e.g. GitHub). See the Nature Portfolio guidelines for submitting code & software for further information.

Data

Policy information about availability of data

All manuscripts must include a data availability statement. This statement should provide the following information, where applicable:

- Accession codes, unique identifiers, or web links for publicly available datasets
- A description of any restrictions on data availability
- For clinical datasets or third party data, please ensure that the statement adheres to our policy

The data that support the findings of this study are available from the corresponding author upon reasonable request. Source files for immunoblot and protein gels are provided with this paper in the Source Data file. In the current study we used the databases PhosPhAt 4.0 [https://phosphat.uni-hohenheim.de/], Uniprot [https://www.uniprot.org], TAIR [https://www.arabidopsis.org/] and the Proteomics DB [https://www.proteomicsdb.org/proteomicsdb/].

Human research participants

Policy information about studies involving human research participants and Sex and Gender in Research.

Reporting on sex and gender	*Use the terms sex (biological attribute) and gender (shaped by social and cultural circumstances) carefully in order to avoid confusing both terms. Indicate if findings apply to only one sex or gender; describe whether sex and gender were considered in study design whether sex and/or gender was determined based on self-reporting or assigned and methods used. Provide in the source data disaggregated sex and gender data where this information has been collected, and consent has been obtained for sharing of individual-level data; provide overall numbers in this Reporting Summary. Please state if this information has not been collected. Report sex- and gender-based analyses where performed, justify reasons for lack of sex- and gender-based analysis.*
Population characteristics	*Describe the covariate-relevant population characteristics of the human research participants (e.g. age, genotypic information, past and current diagnosis and treatment categories). If you filled out the behavioural & social sciences study design questions and have nothing to add here, write "See above."*
Recruitment	*Describe how participants were recruited. Outline any potential self-selection bias or other biases that may be present and how these are likely to impact results.*
Ethics oversight	*Identify the organization(s) that approved the study protocol.*

Note that full information on the approval of the study protocol must also be provided in the manuscript.

Field-specific reporting

Please select the one below that is the best fit for your research. If you are not sure, read the appropriate sections before making your selection.

☒ Life sciences ☐ Behavioural & social sciences ☐ Ecological, evolutionary & environmental sciences

For a reference copy of the document with all sections, see nature.com/documents/nr-reporting-summary-flat.pdf

Life sciences study design

All studies must disclose on these points even when the disclosure is negative.

Sample size	Sample size calculation was not performed prior to the experiment. The sample size was determined based on experience of similar assays performed earlier. The experiments were repeated with at least two biological replicates.
Data exclusions	Root length analyses: Seeds that were not germinated and seedlings that arrested growth after germination were excluded from all root measurements.
Replication	All controls and samples in a single experiment were handled at the same time under the same conditions. All biochemical and molecular biology experiments were conducted at least three times with consistent results. The FRET-based di-UB DUB assay as well as qRT-PCR analysis were performed at least twice with four technical replicates and showed consistent results. Confocal studies on PMA-GFP-UB expressing protoplasts were performed at least three times for each plasmid combinations. The treatment of PIN2-GFP-expressing seedlings BFA, WM, and dark in wild-type and otu11otu12 background was performed at least two times with consistent results. All root length assays were conducted at least two times. All replicates of root length assays and confocal assays had equal results.
Randomization	No randomization was applicable since there was no organization in experimental groups.
Blinding	Blinding was not performed since there was no organization in experimental groups. However, the analysis of experimental data (in vitro DUB assays, lipid binding assays, confocal microscopy with seedlings after BFA, WM and dark treatment, protoplast assays) was performed by at least two persons independently and consistent results were obtained.

Reporting for specific materials, systems and methods

We require information from authors about some types of materials, experimental systems and methods used in many studies. Here, indicate whether each material, system or method listed is relevant to your study. If you are not sure if a list item applies to your research, read the appropriate section before selecting a response.

Materials & experimental systems

n/a	Involved in the study
	x Antibodies
x	Eukaryotic cell lines
x	Palaeontology and archaeology
x	Animals and other organisms
x	Clinical data
x	Dual use research of concern

Methods

n/a	Involved in the study
x	ChIP-seq
x	Flow cytometry
x	MRI-based neuroimaging

Antibodies

Antibodies used

primary antibodies

anti-GST (rabbit) polyclonal, generated for this study (Eurogentech), 1000 x diluted
anti-H+-ATPase (rabbit), polyclonal, Agrisera, AS07260 (lot 1610), 5000 x diluted
anti-UGPase (rabbit), polyclonal, Agrisera, AS05086 (lot 1807), 3000 x diluted
anti-Sec21p (rabbit), polyclonal, Agrisera, AS08327 (lot 1204), 1000 x diluted
anti-GFP (rat) [3H9], monoclonal, Chromotek, 3H9-100 (multiple lots were used including 80626001AB), 1000 x diluted
anti-RFP (mouse) [GT1610], monoclonal, Sigma-Aldrich, SAB2702202 (lot 41169), 1000 x diluted
anti-UB (mouse) [P4D1], monoclonal, Santa Cruz, sc-8017 (multiple lots were used including A1420), 500 - 1000 x diluted
anti-Actin (mouse), [JLA20], monoclonal, Sigma-Aldrich, MABT219 (multiple lots were used over time), 50 x diluted
anti-FLAG (mouse) [M2], monoclonal, Sigma-Aldrich,F1804, 1000 x diluted
anti-OTU11 (rabbit), polyclonal, generated for this study (Eurogentech), 1000 x diluted

secondary antibodies

anti-rat-HRP (goat), Roche, A9037 (lot SLCF6775), 80.000 x diluted
anti-mouse-HRP (rabbit), Sigma-Aldrich, A9044 (multiple lots were used including 106M4870V), 80.000 x diluted
anti-rabbit -HRP (goat), Sigma-Aldrich, A0545 (multiple lots were used including 017M4850V), 80.000 x diluted
anti-mouse lgG, Dylight 488 (goat), Thermo Fisher Scientific, 35503 (lot VB2833224), 5000 x diluted
anti-rabbit lgG, Dylight 650 (goat),Thermo Fisher Scientific, 84546 (lot TA2507602), 5000 x diluted

Validation

Validation statements (URL) for primary antibodies:

Primary antibodies were verified by comparison with the molecular weight marker and comparison with appropriate negative controls. All antibodies that were not generated for this study were used as recommended by the manufacturer. The anti-GST and anti-OTU11 antibody that were generated in this study were used as specified in the Methods section and the Reporting Summary.

anti-GST (rabbit), polyclonal, generated for this study (Eurogentech)
The generation of the antibody is described in the "Methods" section of the manuscript. The specificity of the antibody was validated using recombinant GST-, MBP-, and His-Tag proteins as weil as total plant extracts of wild-type and GFP-OTU11 overexpressing seedlings.

anti-H+-ATPase (rabbit) polyclonal, Agrisera, AS07260 (Datasheet of the product is available under the following link: [https ://www. agrisera.com/ cgi-bin/ibutik/SkapaFaktura.pl?SkrivPDF=J&artnr=AS07%20260&Friendly=hatpase-plasma-membrane-hatpase&skrivpdf=j&Friendly_ Grupp=&funk=visa_artikel&Sprak= EN&artgrp=43]

anti-UGPase (rabbit), polyclonal, Agrisera, AS05086 (Datasheet of the product is available under the following link: [https ://www. agrisera.com/ cgi-bin/ibutik/SkapaFaktura.pl?SkrivPDF=J&artn=AS05%20086&skrivpdf=j&Friendly=ugpase-udp-glu cosepyrophosphorylase-marker -of-cytoplasm&funk=visa_artikel&Sprak= EN&artgrp=9]

anti-Sec21p (rabbit), polyclonal, Agrisera, AS08327 (Datasheet of the product is available under the following link: [https ://www. agrisera.com/ cgi-bin/ibutik/SkapaFaktura.pl?SkrivPDF=J&artn=AS08% 20327&artgrp=67&Sprak=EN&funk=visa_artikel&Friendly_Grupp=&skrivpdf=j&Friendly=sec21p-gamma-subunit-cop-vesicles]

anti-GFP (rat) [3H9], monoclonal, Chromotek, 3H9-100 (Datasheet of the product is available under the following link: [https://www.chromotek.com/fileadmin/content/Images/ Antibodies lgGs/GFP_3H9/029762_ validation_report_ Chromotek.pdf]

anti-RFP (mouse) [GT1610], monoclonal, Sigma-Aldrich, SAB2702202 (Validation by immunoblot. Data available on manufacturer's website: [https://www. sigmaaldrich.com/DE/en/product/sigma/sab2702202]

anti-UB (mouse) [P4D1], monoclonal, Santa Cruz, sc-8017 (Datasheet of the product is available under the following link: [https://datasheets.scbt.com/sc-8017 .pdf]

anti-actin (mouse) [JLA20], monoclonal, Sigma-Aldrich, MABT219 (Validation statement of the product is available under the following link: [https://www.merckmillipore.com/DE/de/product/Anti-Actin-Antibody-clone-JLA20,MM_NF-MABT219#overview "clone JLA20 validated for use in Western Blotting, Immunofluorescence."]

anti-FLAG (mouse) [M2], monoclonal, Sigma-Aldrich, F1804 (Datasheet of the product is available under the following link: [https://www.sigmaaldrich.com/specification-sheets/469/360/F1804-5MG ___ S1GMA _ .pdf]

anti-OTU11 (rabbit), polyclonal, generated for this study (Eurogentech)
The generation of the antibody is described in the "Methods" section of the manuscript. The specificity of the antibody was validated using recombinant GST-OTUll and GST-OTU12 as weil as total plant extracts of wild-type and GFP-OTU11 overexpressing seedlings.
